# Bispecific T cell-engager targeting oncofetal chondroitin sulfate induces complete tumor regression and protective immune memory in mice

**DOI:** 10.1186/s13046-023-02655-8

**Published:** 2023-04-28

**Authors:** Nanna Skeltved, Mie A. Nordmaj, Nicolai T. Berendtsen, Robert Dagil, Emilie M. R. Stormer, Nader Al-Nakouzi, Ke Jiang, Alexandra Aicher, Christopher Heeschen, Tobias Gustavsson, Swati Choudhary, Ismail Gögenur, Jan P. Christensen, Thor G. Theander, Mads Daugaard, Ali Salanti, Morten A. Nielsen

**Affiliations:** 1 Centre for Medical Parasitology, Department of Infectious Diseases, University of Copenhagen and, Copenhagen University Hospital, Copenhagen, Denmark; 2 Vancouver Prostate Centre, University of British Columbia, Vancouver, Canada; 3 Center for Single-Cell Omics and Key Laboratory of Oncogenes and Related Genes, Shanghai Jiao Tong University School of Medicine, Shanghai, China; 4 Precision Immunotherapy, Graduate Institute of Biomedical Sciences, China Medical University, Taichung, Taiwan; 5 Pancreatic Cancer Heterogeneity, Candiolo Cancer Institute - FPO – IRCCS, Candiolo (Torino), Italy; 6 Var2 Pharmaceuticals ApS, Copenhagen, Denmark; 7 Department of Clinical Medicine, University of Copenhagen and Center for Surgical Science, Zealand University Hospital, Copenhagen, Denmark; 8 Department of Immunology and Microbiology, Faculty of Health and Medical Sciences, University of Copenhagen, Copenhagen, Denmark

**Keywords:** Immunotherapy, Cancer, Bispecific antibodies, Targeted therapy, VAR2CSA, Checkpoint inhibitor, T cells therapy, T cell memory

## Abstract

**Background:**

The malaria protein VAR2CSA binds oncofetal chondroitin sulfate (ofCS), a unique chondroitin sulfate, expressed on almost all mammalian cancer cells. Previously, we produced a bispecific construct targeting ofCS and human T cells based on VAR2CSA and anti-CD3 (V-aCD3^Hu^). V-aCD3^Hu^ showed efficacy against xenografted tumors in immunocompromised mice injected with human immune cells at the tumor site. However, the complex effects potentially exerted by the immune system as a result of the treatment cannot occur in mice without an immune system. Here we investigate the efficacy of V-aCD3^Mu^ as a monotherapy and combined with immune checkpoint inhibitors in mice with a fully functional immune system.

**Methods:**

We produced a bispecific construct consisting of a recombinant version of VAR2CSA coupled to an anti-murine CD3 single-chain variable fragment. Flow cytometry and ELISA were used to check cell binding capabilities and the therapeutic effect was evaluated in vitro in a killing assay. The in vivo efficacy of V-aCD3^Mu^ was then investigated in mice with a functional immune system and established or primary syngeneic tumors in the immunologically “cold” 4T1 mammary carcinoma, B16-F10 malignant melanoma, the pancreatic KPC mouse model, and in the immunologically “hot” CT26 colon carcinoma model.

**Results:**

V-aCD3^Mu^ had efficacy as a monotherapy, and the combined treatment of V-aCD3^Mu^ and an immune checkpoint inhibitor showed enhanced effects resulting in the complete elimination of solid tumors in the 4T1, B16-F10, and CT26 models. This anti-tumor effect was abscopal and accompanied by a systemic increase in memory and activated cytotoxic and helper T cells. The combined treatment also led to a higher percentage of memory T cells in the tumor without an increase in regulatory T cells. In addition, we observed partial protection against re-challenge in a melanoma model and full protection in a breast cancer model.

**Conclusions:**

Our findings suggest that V-aCD3^Mu^ combined with an immune checkpoint inhibitor renders immunologically “cold” tumors “hot” and results in tumor elimination. Taken together, these data provide proof of concept for the further clinical development of V-aCD3 as a broad cancer therapy in combination with an immune checkpoint inhibitor.

**Supplementary Information:**

The online version contains supplementary material available at 10.1186/s13046-023-02655-8.

## Background

T cell-engaging bispecific antibodies (T-bsAbs) show growing potential for targeted treatment of cancer. T-bsAbs direct T cells to the tumor to mediate killing of cancer cells, bypassing the need for endogenous tumor-antigen specificity [1–4]. Besides triggering tumor cell death without the need for co-stimulation, the T cell activation and proliferation potentially promote a systemic, tumor-specific immune response [5–7].

More than 40 different T-bsAbs are currently in clinical development for the treatment of hematologic malignancies as well as solid tumors, and targeting different tumor-associated antigens (TAAs) [4, 8, 9]. An ideal TAA is exclusively and abundantly expressed on cancer cells, while absent in healthy tissues. During our research on pregnancy-associated malaria such a TAA was discovered. A unique chondroitin sulfate glycosaminoglycan expressed as proteoglycans (CSPGs) on the syncytiotrophoblast epithelial placental tissue, is also present on the vast majority of cancer cell lines and cancer tissues [10–12]. This unique oncofetal chondroitin sulfate (ofCS) is omnipresent in malignancies, indicating an essential role in tumorigenesis. Indeed, several studies have reported increased levels of abnormally expressed chondroitin sulfate (CS) across various cancers, which correlates with poor prognosis [13–16]. CS is likewise found to inhibit TNF-α-induced NF-κB activation and downstream inflammatory processes [17, 18]. Due to the cancer cell restricted distribution of ofCS and possible essential role in tumorigenesis, ofCS provides a promising target.

OfCS can be specifically targeted by the malaria protein VAR2CSA, which enables sequestration of *Plasmodium falciparum*-infected erythrocytes in the placenta [12, 19–21]. Recently, a recombinant sub-fragment consisting of the minimal binding region of VAR2CSA (rVAR2) [22, 23] was combined in a human T cell-engaging bispecific molecule (V-aCD3^Hu^). V-aCD3^Hu^ demonstrated efficacy in a xenograft bladder cancer model in immunocompromised mice with human peripheral blood mononuclear cells (PBMCs) injected locally [24].

Despite providing valuable insights, immunodeficient xenograft models cannot fully recapitulate the complexity of long-term tumor immunity due to graft-versus-host responses and poor survival of the human PBMCs [25, 26]. By using a syngeneic tumor model in mice with a functional immune system we were able to investigate immune cell activation, infiltration, and memory, as was done for several immune checkpoint inhibitors (ICIs) [27, 28]. Furthermore, the syngeneic models allow for the full effect of potential antigens created by the tumor (neoantigens) released by T-bsAb-mediated cancer cell cytotoxicity, which correlate with mutational load and, as a result, ICI function [29–31]. As ofCS is expressed on nearly all mammalian cancer cells [12], we were able to use mice with syngeneic tumors and a fully functioning immune system to investigate the systemic, local, and long-term anti-tumor effects of V-aCD3^Mu^. V-aCD3^Mu^ consists of the ofCS-binding rVAR2 linked to a single-chain variable fragment derived from an anti-murine CD3 antibody (aCD3^Mu^) [32].

Due to the immunomodulating features of the TAA ofCS [33], we hypothesize that V-aCD3^Mu^ treatment, besides increasing neoantigen presentation through cancer cell killing, also increases immune cell infiltration into the tumor. Since a “cold” immune-phenotype is known to prevent or lower the effects of ICIs, we anticipate an increase in the effects of these when given in combination with V-aCD3^Mu^. In this study, we show the efficacy of V-aCD3^Mu^ as a single treatment and in combination with an ICI or the innate immune-stimulating CpG-oligodeoxynucleotides (CpG) [34]. This was done using the syngeneic tumor models for immunologically “cold” tumors 4T1 mammary carcinoma and B16-F10 malignant melanoma, and the immunologically “hot” CT26 colon carcinoma model [35]. All three are tumor models where the effect of the ICI alone has potential for improvement.

## Materials and methods

### Study design

This study was initiated to test short- and long-term effects of V-aCD3^Mu^ on cancer in mice with a competent immune system, to potentially make V-aCD3^Hu^ an interesting therapy against human cancers. Mice were randomly assigned or grouped based on tumor size for similar means. Treatments and measurements were blinded except in the intratumoral and splenic cell subset experiments and the rechallenge studies. Sample sizes were chosen based on power estimations. Sample sizes were based on the three Rs and previous results. Data collection was stopped at pre-defined timepoints or when reaching a terminal endpoint.

### Cell culturing

4T1, CT26, TC-1 cancer cell lines, and mouse splenocytes were maintained in RPMI 1640 (Sigma Aldrich), while B16-F10 were grown in DMEM Glutamax (Gibco) and kept at 3s7ºC in a humidified atmosphere of 5% CO2. Media was supplemented with 10% fetal bovine serum, 1% penicillin/streptomycin, and 1% L-glutamine if not supplemented from manufacturer. TC-1 cell media was additionally supplemented with 1% non-essential amino acids and 1% L-pyruvate.

For in vivo experiments, cells were thawed from our cell bank and passaged 3–4 times before injection at 40–80% confluency. For in vitro experiments, cells were cultured for up to 20 passages.

Murine PDAC cells were derived from genetically engineered and fully backcrossed KPC mice (LSL-Kras^G12D/+^; p53^f/f^; Pdx1-Cre). Pancreatic tumor cells from KPC mouse CHX2000 were obtained by dissociating pancreatic tumor pieces in a Miltenyi dissociator after incubation in collagenase I (STEMCELL Technologies) for 20 min. Cells were cultured and passaged in F12/DMEM (Thermo Fisher Scientific) (1:1) supplemented with 5% fetal bovine serum (FBS) + 1% penicillin/streptomycin. CHX2000 were transduced with a pLVX-Luc2-P2A-AcGFP1-puro Lentivector system (Hedgehobio, HH-LV-056, China) for in vivo tracking of bioluminescence. Forty-eight hours prior to injection, the maintenance medium was replaced by macrophage-conditioned medium to induce epithelial-mesenchymal-transition for enhanced metastasis.

Mycoplasma testing was routinely performed every six weeks by PCR on culture medium from cells grown for at least 24 h.

4T1, B16-F10, CT26, and TC-1 were kindly provided by our collaborators at Vancouver Prostate Centre, British Columbia, Canada, the Clausen group, Department of Cellular and Molecular Medicine, University of Copenhagen, Denmark, and CureVac, Tübingen, Germany.

### Preparation of macrophage-conditioned medium (MCM)

Monocyte-derived human macrophage cultures were produced from PBMCs obtained from healthy donors. PBMCs were cultured in IMDM (Gibco) supplemented with 10% human AB serum (Sigma-Aldrich)(IMDM +). The next day, adherent PBMC-derived monocytes were polarized with 0.5 ng/ml of M-CSF (PeproTech) in IMDM + for 48 h to induce ‘M2-tumor associated-like’ macrophages. Macrophages were washed with PBS and cultured in DMEM/F12 supplemented with 2% B-27, 2 mM L-Glutamine, 50 U/mL penicillin/streptomycin, and 20 ng/mL FGF for 48 h. Supernatants were spun to remove remaining cells.

### Isolation and activation of splenocytes

Spleens from naïve mice were placed in RPMI 1640, disrupted with a syringe plunger, and filtered through a Falcon 40 µm cell strainer (Corning). Splenocytes were kept at 2–8 million cells/mL for four days in RPMI 1640 media supplemented with 4 μg/mL Concanavalin A from Canavalia ensiformis (Sigma-Aldrich) and 0.24 or 1.2 ng/mL human recombinant IL-2 (CHO-expressed, Stemcell Technologies) if not used immediately. T cell isolation from splenocytes was performed by negative bead selection using the EasySep™ Mouse T cell Isolation Kit (Stemcell Technologies).

### Protein production

Recombinant VAR2CSA (rVAR2) was produced in SHuffle T7 *E. coli* cells as either SpyTag-DBL1-ID2a (121 kDa) or SpyTag-ID1-ID2a (70 kDa) including a C-terminal -6xHIS-tag and a V5-tag, as previously described [12]. Anti-murine CD3-SpyCatcher (aCD3^Mu^) was produced as a single-chain variable fragment using the sequence from Gilliland et al. [32]. The anti-CD3 (40 kDa) construct was genetically fused to SpyCatcher and expressed in *E. coli* BL21 cells as inclusion bodies. Purification by refolding was performed by redissolving the pellet in denaturing buffer (8 M urea, 20 mM Tris–HCl pH 8.0, 500 mM NaCl, 5 mM DTT) followed by centrifugation at 40,000 g for 60 min. The supernatant was incubated with Ni–NTA agarose beads overnight. Next, eluted protein was refolded by dialysis toward dialysis buffer (3 M urea, 50 mM Tris–HCl pH 8.0, 150 mM NaCl, 3 mM GSH/1 mM GSSH) and after 24 h extensively dialyzed toward PBS pH 7.4. Monomeric aCD3^Mu^ was isolated by IMAC (HisTrap HP, Cytiva) and buffer exchanged into PBS pH 7.4.

The genetically fused V-aCD3^Mu^ (V-aCD3^Mu*^) consisted of the single-chain variable fragment of aCD3^Mu^ followed by a GSAGGSGGDS linker between rVAR2 ID1-ID2a with a V5-tag and a Streptag II. This 97 kDa protein was expressed in baculovirus-infected Sf9 insect cells and purified using a streptactin XT column (IBA-Lifesciences) according to manufacturer’s protocol.

Protein purity was assessed by running 1 µg on an SDS-PAGE 4–12% BIS/TRIS with and without reducing agent (DTT) after boiling. The gel was stained in InstantBlue Coomassie protein stain before imaging.

### Protein binding in flow cytometry

For binding assays, cancer cells were detached using CellStripper (Corning) and spleen or blood was freshly harvested from a BALB/c mouse. Red blood cells were lysed from whole blood by a 13 min. incubation in red blood cell lysis buffer containing 0.155 M ammonium chloride, 0.01 M potassium hydrogen carbonate, and 0.1 mM EDTA.

Cells were washed in PBS with 2% FBS before each incubation and kept on ice throughout. First incubation was with relevant proteins with/without 0.5 mg/mL CSA (Sigma) for 30 min, followed by incubation with anti-V5 (FITC, Invitrogen) or anti-penta-HIS (Alexa Fluor 488, Qiagen) antibodies, together with anti-CD4 (GK1.5, APC/Cy7, BioLegend) and anti-CD8 (53–6.7, APC, BD Biosciences) if detecting T cells. All flow cytometry was analyzed using LSR-II, Fortessa-3, or -5 (BD Biosciences) immediately, or the next day after cell fixation in 4% paraformaldehyde.

### In vitro killing assay

Five thousand luciferase transfected or regular cancer cells were seeded in each well of a flat-bottomed black (Nunc) or transparent (ThermoFisher) 96-well plate, respectively, and incubated overnight in 37ºC in a humidified atmosphere of 5% CO2. Splenocytes/T cells from C57BL/6 mice were added to each well at an effector:target ratio of 10:1. Cytotoxicity was measured by luciferase activity using the SpectraMax i3x (Molecular Devices)/Perkin Elmer TopCount NXT or crystal violet staining using the HiPo MPP-96 microplate photometer (Biosan). Supernatants were removed before measuring cytotoxicity and saved at -80°C for subsequent analysis.

### Mice and treatments

6–8°weeks old C57BL/6 J and BALB/c AnNRJ (BALB/c) mice (Janvier labs) were kept for at least one week prior to use. 100 µL PBS with B16-F10 (100,000), 4T1 (75,000), or CT26 (500,000) cells were injected subcutaneously (SQ) in the lower left quadrant of the belly or in the flank. SQ tumors were peritumorally treated with 12 μg V-aCD3^Mu^ (coupled) or 15 μg V-aCD3^Mu^ (fused) (equal molar amounts) in PBS (50 μL in total). 100 μg murine anti-mCTLA-4 (Invivofit) or InvivoPlus anti-mouse PD-1 (BioXCell) was intraperitoneally administered three times within the first week with a double first dose, or twice within the first week, respectively. 50 μg CpG-ODN 1668 (Invivogen) was administered on day 5 peritumorally followed by 20–30 μg when indicated. Tumors were measured using a caliper tool and volume was calculated according to the formula $$\mathrm{V}=0.5*\mathrm{length}*{(width)}^{2}$$.

Tramadol (20 mg/kg) was injected SQ 15 min before surgery. Following anesthesia (Isoflurane 2%), the orthotopic PDAC was created by injecting 5 × 10^4^ CHX2000 PDAC-Luc cells in 40 μl of diluted Matrigel (Corning, 356,234) (Cells:Matrigel = 1:1) through an incision into the middle of the pancreas of 6-week-old C57BL/6 mice (Lingchang-bio, China). The abdominal wall was sutured, and the skin stapled. Mice were randomized on day 8 for treatments day 8, 11, 14, 32, 35, and 38 after implantation. Following anesthesia and careful palpation of the pancreatic tumors, 15 µg V-aCD3^Mu^/0.1 ml PBS or PBS was transdermally injected around the tumor. Tumor sizes were monitored by luminescent imaging (IVIS-200, PerkinElmer) 20 min after injecting anesthetized mice with 150 mg/kg luciferin. Luciferase activity is displayed as total flux measured as luminescence in photons per second.

All animal experiments using 4T1, B16-F10, and CT26 were performed at the Department for Experimental Medicine at University of Copenhagen, Denmark in accordance with the FELASA Rodent Health Surveillance program and approved by the Animal Experiments Inspectorate (P19-117 and P21-119). Experiments employing CHX2000 were performed at Shanghai Jiao Tong University and were approved by the local Animal Care and Use Committee (Shanghai Jiao Tong University Project Approval A-2021–001).

### T cell activation and memory in spleens, tumors, and LNs

After B16-F10 injection in the right flank, the C57BL/6 J mice received V-aCD3^Mu^ (day 6, 11, and 13) and aCTLA-4 (day 6, 9, and 11). Spleens and tumors were taken out 14 days after tumor injection and put in supplemented RPMI or DMEM, respectively. The organs were mechanically disrupted, filtered through a cell strainer, and washed in PBS with 2% FBS. The splenocytes were counted to calculate the total cell number. After Fc block (CD16/CD32 mouse, BD Biosciences), cells were stained for 15 min with Zombie Aqua™ Fixable viability marker, washed and incubated with antibodies against CD3 (17A2, PerCP/Cy5.5), CD4 (GK1.5, APC/Cy7), CD69 (H1.2F3, BV650), CD25 (PC61, BV786), CD49b (DX5, PE/Cy7), CD49d (9C10 (MFR4.B), BV711), CD8 (53–6.7, APC), and CD44 (IM7, Pacific blue) from BioLegend/BD Biosciences/Invitrogen. The cells were fixed, permeabilized, and intracellularly stained using the FoxP3/ transcription factor staining buffer set (eBioscience) and a FoxP3 antibody (MF-14, PE, Biolegend) according to the manufacturer’s instruction before measuring fluorescence.

BALB/c mice recovered from 4T1/CT26 tumors were sacrificed 120 days or 157 days, respectively, after tumor injection (age 6/7 months) along with naïve control mice (age 2 months). Right inguinal lymph nodes (LNs) and spleens were put in supplemented RPMI media. Next, the organs were prepared, stained, and analyzed as described above.

### Patient and public involvement

Patients were not involved in this study.

### Data Analysis and statistics

Nonparametric tests were used for sample sizes smaller than 10. For comparison between two groups, the Mann–Whitney test was used, while more groups were compared using the Kruskal–Wallis test with Dunn’s post hoc test. Simple linear regressions were used to evaluate the relationship between cell types and tumor size.

One-way ANOVA was used for sample sizes of 10 or more after confirmation of normal distribution through QQ-plots. Groups were compared to the PBS group and multiple comparisons were adjusted for using Dunnett’s post hoc correction. The testing level α = 0.05 was used for all statistics.

Flow cytometry data were analyzed using FlowJo™ v10.8.1 Software (BD Life Sciences) and serum cytokine levels were analyzed by Discovery Workbench 4.0 (Mesoscale discoveries). Figures and statistical analysis were made using GraphPad Prism version 9.3.0, GraphPad Software, San Diego, California USA, www.graphpad.com.

## Results

### rVAR2 coupled to aCD3^Mu^ retains cancer cell binding

**Fig. 1:**
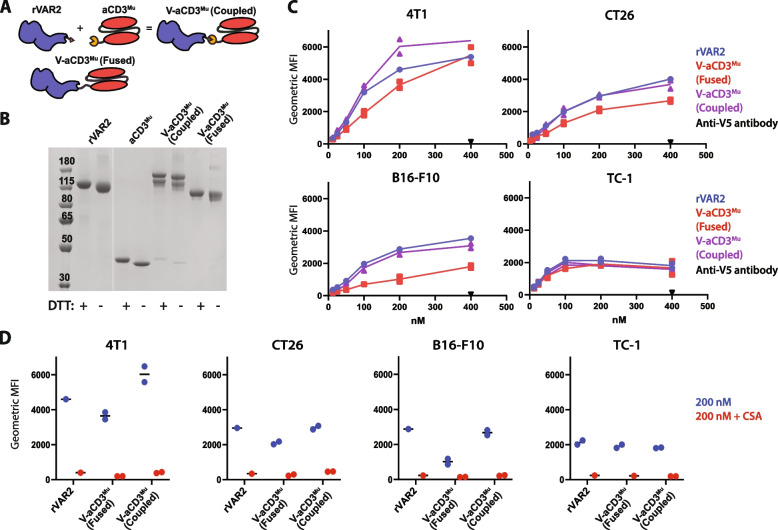
rVAR2 coupled to aCD3^Mu^ retains cancer cell binding. **A** Schematic figure of rVAR2 and aCD3^Mu^ conjugated through the SpyTag/SpyCatcher system into one protein (V-aCD3^Mu^ (coupled)) very similar to the genetically fused V-aCD3^Mu^. **B** SDS-PAGE of rVAR2, aCD3^Mu^, V-aCD3^Mu^ (coupled), and V-aCD3^Mu^ (fused). **C** Flow cytometry showing binding of rVAR2, V-aCD3^Mu^ (coupled), and V-aCD3^Mu^ (fused) to the indicated cancer cell lines, including the detection antibody anti-V5 as a control. **D** Flow cytometry showing binding of 200 nM of indicated protein with and without soluble chondroitin sulfate A (CSA) added in excess. Each dot represents one data point. TC-1 binding of V-aCD3^Mu^ (coupled) and V-aCD3^Mu^ (fused) were evaluated in individual experiments and values were normalized relative to rVAR2 binding ((V-aCD3^Mu^ (coupled)/rVAR2_1) * VAR2_2). Data are representative of either two (B16-F10 and TC-1) or four (4T1 and CT26) individual experiments

We have previously shown that rVAR2 binds the vast majority of cancer cell lines, including murine cancers [12]. This served as benchmark for assessing whether the binding capability of rVAR2 changes when conjugated to murine anti-CD3 (aCD3^Mu^) using the SpyTag/SpyCatcher system (V-aCD3^Mu^ (coupled)) [36] or when genetically fused (V-aCD3^Mu^ (fused)) (Fig. 1A, Fig. 1B). For a head-to-head comparison of the two bispecific proteins, their binding capabilities were evaluated using ELISA and flow cytometry (Fig. 1C, Fig. S1A).

Both V-aCD3^Mu^ constructs bound the four different murine cancer cell lines and CSPG, although minor difference was observed (Fig. 1C, Fig. S1A). The binding of V-aCD3^Mu^ (coupled) to cancer cells was comparable to the binding of rVAR2, while there was a tendency towards a lower binding of V-aCD3^Mu^ (fused) for the B16-F10 cell line (Fig. 1C). Addition of soluble chondroitin sulfate completely inhibited the cancer cell binding to all cell lines, demonstrating the specificity of rVAR2 (Fig. 1D). As V-aCD3^Mu^ (coupled) and V-aCD3^Mu^ (fused) also have similar effects in vivo (Fig. S1B), they are interchangeably referred to as V-aCD3^Mu^ in the text, while in the figures an asterisk marks the genetically fused compound.

### aCD3^Mu^ retains T cell binding when linked to rVAR2

**Fig. 2:**
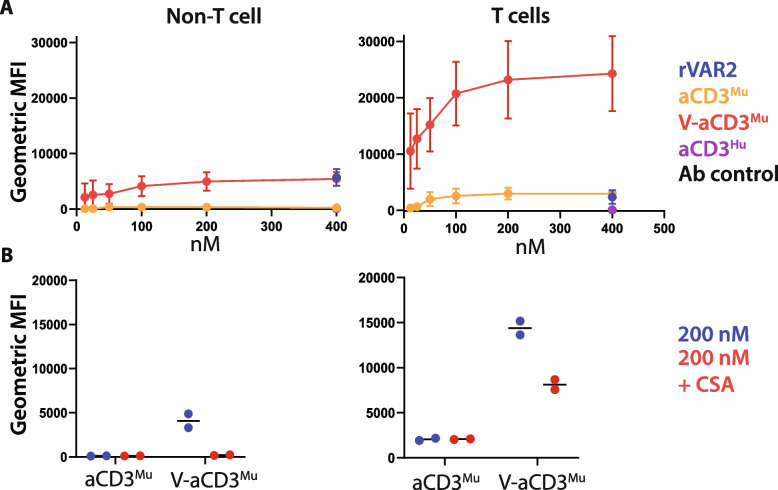
aCD3^Mu^ retains T cell binding when linked to rVAR2. **A** Flow cytometry on protein binding to non-T cells (CD4-CD8-) and T cells (CD4 + /CD8 + /CD4 + CD8 +) from splenocytes and white blood cells. An anti-human anti-CD3 (aCD3^Hu^) and an antibody control (anti-CD8, anti-CD4, and the secondary antibody anti-penta-HIS) were included as controls. Means and standard deviations are displayed. **B** CSA inhibition of protein binding to non-T cell splenocytes/white blood cells (left panel) and T cells (right panel). Each dot represents one data point. Data in this figure is compiled from three separate experiments. Note that the fluorescent signals cannot be directly compared as V-aCD3^Mu^ has two penta-HIS tags while aCD3^Mu^, rVAR2, and aCD3^Hu^ only have one

To examine aCD3^Mu^ binding to murine T cells and recombinant murine CD3, we performed flow cytometry and ELISA (Fig. S2A, B). V-aCD3^Mu^ and aCD3^Mu^ both bound T cells and showed no binding to non-T cells when soluble CSA was added (Fig. 2A, Fig. 2B). V-aCD3^Mu^ showed a higher signal in binding to T cells and recombinant CD3 than aCD3^Mu^ alone, which is partly due to V-aCD3^Mu^’s two detection tags, whereas aCD3^Mu^ and rVAR2 only have one.

Based on this, we concluded that the aCD3^Mu^ domain of the V-aCD3^Mu^ protein binds T cells in vitro.

### V-aCD3^Mu^ mediates in vitro killing of cancer cells

**Fig. 3:**
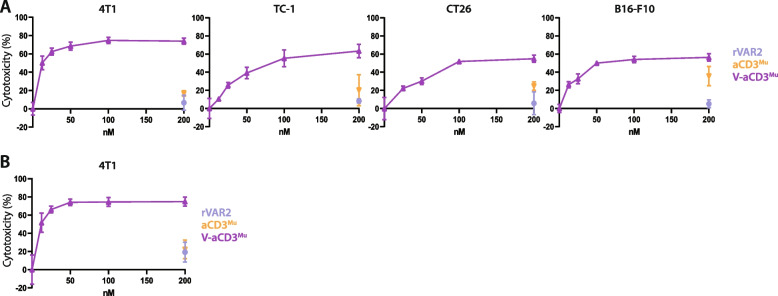
V-aCD3^Mu^ mediates in vitro killing of cancer cells. **A** Killing assays with preactivated splenocytes added in an E:T ratio of 10:1 to either 4T1, CT26, B16-F10, or TC-1 cells together with V-aCD3^Mu^ in a twofold titration series from 200 nM and rVAR2 and aCD3^Mu^ at 200 nM concentrations. **B** Killing assay with isolated T cells as effector cells. The killing assay was performed as described above using 4T1 cells. Cytotoxicity data represents data from four (4T1), two (CT26), or one (B16-F10, TC-1) experiments

To determine whether V-aCD3^Mu^ elicits cytotoxicity against cancer cells, we assessed cell killing after incubation with mouse splenocytes. Splenocytes were pre-incubated with Concanavalin A and IL-2 and added to cancer cells in an effector target ratio (E:T) of 10:1. Target cell viability was assessed after 48 h.rVAR2 alone did not affect cancer cell survival while aCD3^Mu^ increased cytotoxicity in high concentrations, especially against B16-F10 cells. V-aCD3^Mu^, however, elicited a stronger, T cell-mediated dose-dependent killing and IFN-γ secretion exceeding those elicited by rVAR2 or aCD3^Mu^ alone (Fig. 3A, 3B, and S3).

### V-aCD3^Mu^ promotes tumor regression and prevents growth of non-established tumors

**Fig. 4:**
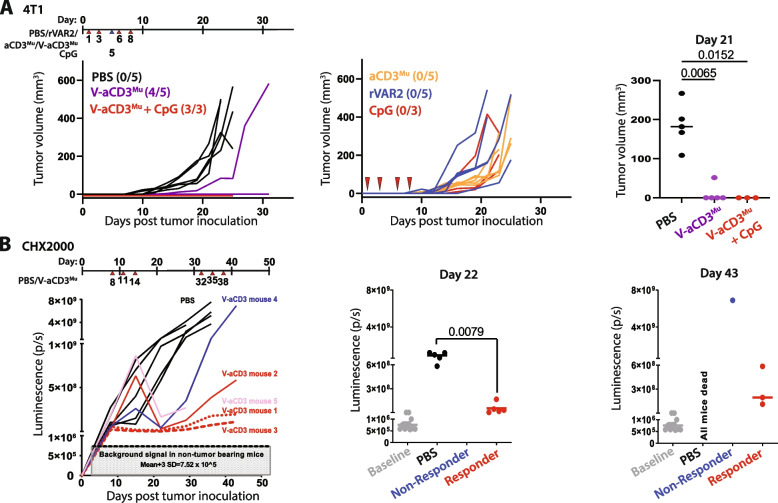
V-aCD3^Mu^ promotes tumor regression and prevents growth of non-established tumors. **A** 4T1 tumors were peritumorally treated on day 1, 3, 6, and 8 after cancer cell inoculation (indicated by red arrows),before the tumors were established. The mice were treated with either PBS (number of mice (*n*) = 5), rVAR2 (*n* = 5), aCD3^Mu^ (*n* = 5), CpG (*n* = 3), V-aCD3^Mu^ (*n* = 5), or V-aCD3^Mu^ + CpG (*n* = 3). CpG was only administered on day 5 (indicated by a blue arrow).Numbers in parentheses indicate the number of tumor-free animals out of all animals in the group. **B** Quantification of bioluminescence in vivo imaging of C57BL/6 mice following orthotopic implantation of 5 × 10^4^ Luciferase^+^ primary pancreatic cancer cells (CHX2000) derived from KPC mice (LSL-Kras^G12D/+^; p53^f/f^; Pdx1-Cre). The mice received intratumoral injections of V-aCD3^Mu^ (*n* = 5) or PBS (*n* = 5) on day 8, 11, 14, 32, 35, and 38. Tumor volumes of treatment versus control group were compared using the Mann–Whitney test. (Left) Tumor growth in individual mice. Baseline luminescence levels of non-tumor-bearing mice are indicated. (Right) Quantification on day 22 and day 43 displays responders and non-responders. On day 22 all mice in the treatment group are responders

We next evaluated the anti-tumor effect of V-aCD3^Mu^ in vivo using mice with fully competent immune systems and syngeneic allografted 4T1 tumors. To test the effect of further boosting the immune system, we included a group that received V-aCD3^Mu^ in combination with CpG. Treatment was initiated one day after cancer cell injection. The mice received four peritumor treatments with two to three days intervals. Mice treated with the control compounds PBS, rVAR2, aCD3^Mu^, or CpG all developed tumors. Four out of five V-aCD3^Mu^-treated and three out of three V-aCD3^Mu^ + CpG-treated mice remained tumor-free and mice in these groups had significantly smaller tumors than mice receiving PBS (*p* = 0.0065 and *p* = 0.015, respectively on day 21, Fig. 4A and S4A).

To further test efficacy in a model with increased heterogeneity and a more realistic tumor microenvironment [37, 38], we employed an orthotopic pancreatic cancer model using cancer cells (CHX2000) derived from genetically modified KPC mice with pancreatic cancer to investigate if V-aCD3^Mu^ was efficacious in mice with established solid tumors. On day 22, we observed statistically significant inhibition of tumor growth in the V-aCD3^Mu^ treatment group (*p* = 0.0079), with a particularly strong effect in three out of five mice (Fig. 4B, S4A, and S4B). On day 43, one mouse receiving V-aCD3^Mu^ treatment did not respond, while three V-aCD3^Mu^-treated mice still demonstrated anti-tumor responses.

### V-aCD3^Mu^ in combination with ICIs eliminates solid tumors in different cancer models

**Fig. 5:**
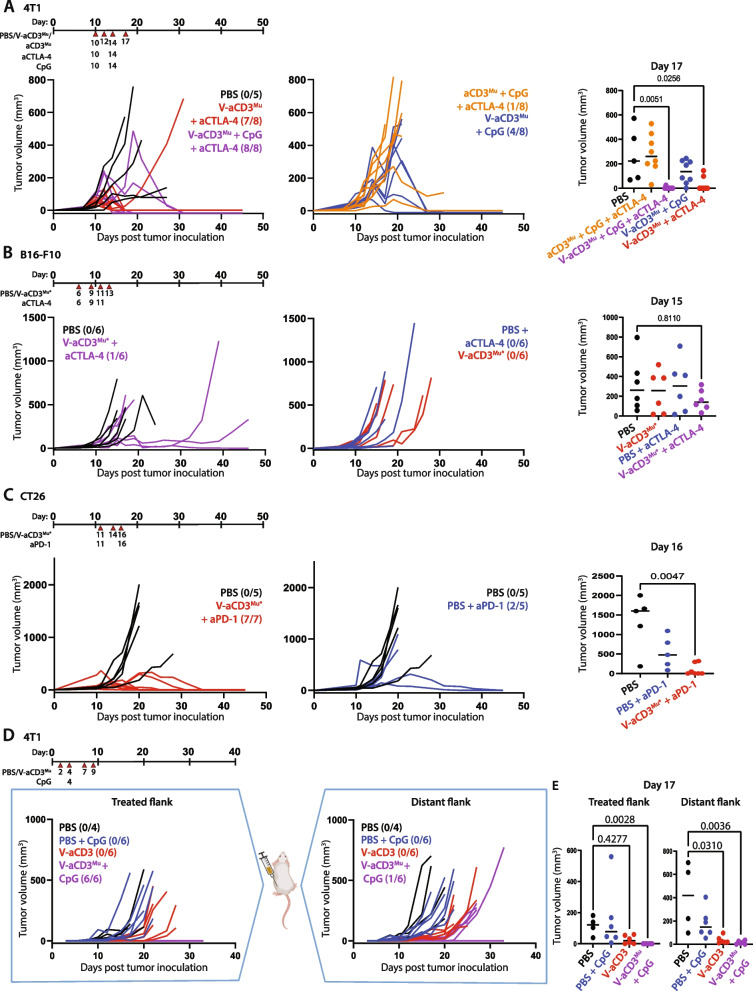
V-aCD3^Mu^ in combination with ICIs eliminates solid tumors in different cancer models. **A** Established 4T1 tumors were treated with either PBS (*n* = 5), CpG + aCTLA-4 + aCD3^Mu^ (*n* = 8), V-aCD3^Mu^ + aCTLA-4 (*n* = 8), V-aCD3^Mu^ + CpG (*n* = 8), or V-aCD3^Mu^ + aCTLA-4 + CpG (*n* = 8) on day 10 (tumor average = 50–100 mm^3^), 12, 14, and 17 as illustrated on the treatment timeline. **B** Established B16-F10 SQ tumors in the left lower quadrant of the abdomen were treated with either PBS (*n* = 6), PBS + aCTLA-4 (*n* = 6), V-aCD3^Mu*^ (*n* = 6), or V-aCD3^Mu*^ + aCTLA-4 (*n* = 6) on day 6 (tumor average = 11 mm^3^), 9, 11, and 13. **C** Established CT26 SQ tumors were treated when they were 106 mm^3^ on average with either PBS (*n* = 5), PBS + aPD-1 (*n* = 5), or V-aCD3^Mu*^ + aPD-1 (*n* = 7) on day 11, 14, and 16, with no aPD-1 given on day 14. **D** Tumor measurements of BALB/c mice inoculated with 4T1 cancer cells in both the left and the right flank. On day 2, 4, 7, and 9, V-aCD3^Mu^ or PBS was injected into the right flank of the mice. CpG was administered mixed in with the treatment on day 4. **E** Tumor measurements of the treated and untreated flanks on day 17 after tumor injection. Numbers in parentheses indicate the number of tumor-free mice or tumor-free flank out of all animals in one group. Tumor volumes were compared to the PBS group using the Kruskal–Wallis test followed by Dunn’s post hoc test

The effect of V-aCD3^Mu^ in solid tumors encouraged us to examine the potential even further in SQ syngeneic models with different immune-infiltration. For this, we used the immunologically “hot” CT26 and the immunologically “cold” B16-F10 and 4T1 cancer tumor models [35]. We tested the effect of V-aCD3^Mu^ in combination with aCTLA-4, CpG, or CpG and aCTLA-4 in established 4T1 tumors. On day 17, mice that received V-aCD3^Mu^ in combination with aCTLA-4 or aCTLA-4 and CpG had statistically significantly smaller tumors than those receiving PBS (*p* = 0.026 and *p* = 0.0051, respectively) and all or almost all mice became tumor-free (Fig. 5A). V-aCD3^Mu^ in combination with aCTLA-4 significantly increased the probability of survival compared to PBS treatment (Fig. S5A). The effect of combining V-aCD3^Mu^ with CpG was less potent, however half of the mice became tumor-free (Fig. 5A).

Next, we assessed the effect of V-aCD3^Mu^ and aCTLA-4 in the B16-F10 tumor model. Half the mice did not respond to the combined treatment and the probability of survival was not significantly altered (Fig. 5B and S5B). However, a third of the mice had a pronounced delay in tumor growth.

In the CT26 colon carcinoma model, V-aCD3^Mu^ was combined with aPD-1. aPD-1 treatment inhibited tumor growth as two out of five animals exhibited ablation of their tumors (Fig. 5C). V-aCD3^Mu^ profoundly enhanced this effect as seven out of seven animals became tumor-free (*p* = 0.0047 on day 16). Both treatments significantly improved survival (Fig. S5C).

To assess whether the treatment with V-aCD3^Mu^ elicits abscopal effects, we injected 4T1 cells in both flanks of the mice. We only initiated peritumoral treatment in the right flank, leaving the left flank untreated. The mice received four treatments of V-aCD3^Mu^ or PBS every 2–3 days from day two after cancer cell inoculation. CpG was given on day four mixed with either PBS or V-aCD3^Mu^. In the treated flank, tumor growth was completely abolished in mice receiving V-aCD3^Mu^ and CpG (*p* = 0.0028, Fig. 5D). As hoped, the tumor development was also significantly inhibited in the untreated flank when treated with V-aCD3^Mu^ alone (*p* = 0.031) or V-aCD3^Mu^ and CpG in combination (*p* = 0.0036) (Fig. 5E).

V-aCD3^Mu^ combined with different ICIs or immune stimulants is more potent than any of the treatments by themselves and the combined treatment even induced abscopal effects on immunologically “cold” solid tumors. Interestingly, aCTLA-4 treatment at this dose level, alone or combined with aCD3^Mu^ and CpG, did not have a significant effect on B16-F10 or 4T1 tumor progression. In combination with V-aCD3^Mu^, aCTLA-4 resulted in full protection in most of the mice. All mice that became tumor-free after treatment stayed tumor-free for at least six months.

### Combination of V-aCD3^Mu^ and ICI leads to increased levels of activated and memory T cells

T-bsAbs are known to recruit T cells to the tumor and cause MHC-unrestricted killing of cancer cells by both CD8 + and CD4 + T cells, which enables neoantigen release and thus a potential expansion of neoantigen-specific T cells [5, 39–42]. To investigate if V-aCD3^Mu^ alone or combined with aCTLA-4 also increased the level of activated and memory T cells, we performed flow cytometry on splenocytes and dissociated tumor from the B16-F10 model where aCTLA-4 had no effect on its own, likely due to the lack of immune infiltration [27, 28]. Tumors and spleens were harvested on day 14, one day after the last treatment for subsequent analysis by flow cytometry (Fig. S6A).

**Fig. 6:**
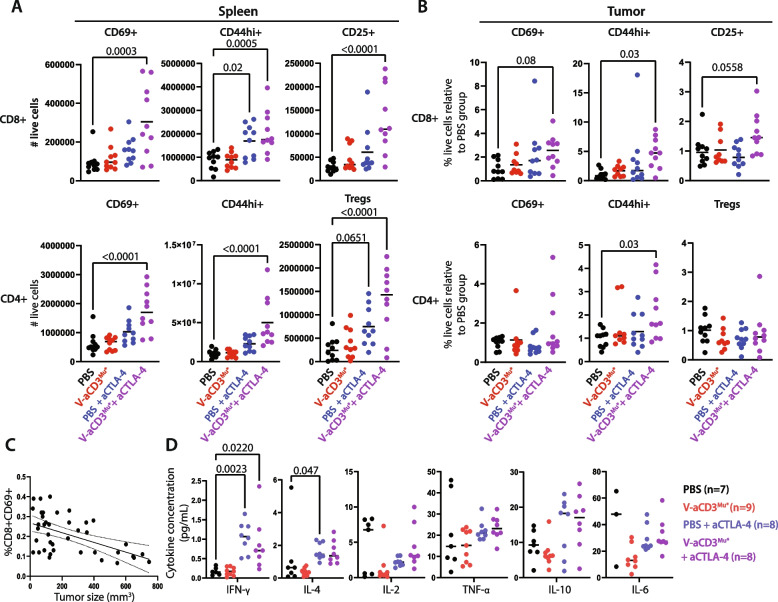
Combination of V-aCD3^Mu^ and ICI leads to increased levels of activated and memory T cells. Data are from flow cytometry on 40 C57BL/6 mice with B16-F10 tumors which were sacrificed on day 14 after tumor injection. The mice received either PBS, V-aCD3^Mu*^, PBS + aCTLA-4, or V-aCD3^Mu*^ + aCTLA-4 on day 6, 9, 11, and 13 after cancer cell injection in two separate experiments with 20 mice in each. **A** Flow cytometry showing total number of splenic CD8 + and CD4 + T cells that were CD69 + , CD44^hi^, or CD25 + (CD25 + FoxP3 + for Tregs). **B** Relative change in % of CD8 + and CD4 + tumor-infiltrating T cells (CD69 + /CD44^hi^/CD25 + /CD25 + FoxP3 +) in comparison to the PBS group. The mean is displayed. **C** Correlation between tumor size and the percentage of CD8 + CD69 + cells of all live single cells from the spleen evaluated by simple linear regression (*p* = 0.0003). **D** Cytokine concentrations from serum measured in an MSD V-plex assay. Statistics from A and B are done using one-way ANOVA with Dunnett’s post hoc test for comparison of treatment groups to PBS group. Statistics from (D) are performed using the Kruskal–Wallis test followed by Dunn’s post hoc test

In the spleen, we observed a statistically significant increase in the number of cytotoxic T cells and T helper cells expressing different activation (CD69 + and CD25 + [43, 44]) and memory (CD44^hi^ [44, 45]) markers in mice receiving V-aCD3^Mu^ combined with aCTLA-4 compared to mice receiving PBS (Fig. 6A)[43–45]. This increase was also evident when considering the percentage of these populations relative to the PBS group (Fig. S6B). In addition, an increase in the percentage and total number of Tregs in the spleen was observed (Fig. 6A + Fig. S6B). The increase in Tregs, effector, and memory T cell splenocytes in the group receiving V-aCD3^Mu^ combined with aCTLA-4 was also evident when comparing UMAPs [46] of T cell clusters between the different treatment groups (Fig. S6C).

In tumor tissue, V-aCD3^Mu^ in combination with aCTLA-4 significantly increased the fraction of CD44^hi^ cytotoxic T cells and T helper cells compared to mice receiving PBS, while the fraction of Tregs did not increase (Fig. 6B).

Together, these findings suggest that V-aCD3^Mu^ and aCTLA-4 in combination have started the cancer-immunity cycle, leading to both systemically activated T cells, and also infiltration of activated T cells back into the tumor.

As the treatment response varied within the combined treatment group in the B16-F10 model, we investigated if the increase in different cell types correlated with the tumor size. A strong correlation between the percentage of activated splenic cytotoxic T lymphocytes (CD8 + CD69 +) of all live cells in the spleen and the tumor size was observed, indicating that this could be a potential measure for therapy response in this model (Fig. 6C). Notably, this was not observed in the tumor. Here, tumor size correlated better with the percentage of activated or resident T helper cells of all live cells (CD4 + CD69 +)(Fig. S6D) [47, 48].

Administration of immunotherapy is often limited by toxicity, as it increases the risk of cytokine storms [41]. Thus, we investigated whether local administration of V-aCD3^Mu^ led to a systemic increase of relevant cytokines. Cytokine levels after V-aCD3^Mu^ treatments were not augmented compared to the PBS control group nor when used in combination with aCTLA-4 administrations (Fig. 6D). This looks promising for the safety profile of intratumor V-aCD3 administration.

Collectively, our data suggest that neither V-aCD3^Mu^ nor aCTLA-4 alone significantly increase the number or percentage of activated T cells. When combined, however, they more potently increase T cell activation systemically in the spleen and locally in the tumor. V-aCD3^Mu^ combined with aCTLA-4 also induced a systemic expansion of activated and memory T helper cells and cytotoxic T cells. The higher percentage of activated T cells correlate with a smaller tumor size, making recovery more likely in mice treated with V-aCD3^Mu^ and aCTLA-4 in combination.

### Recovered mice reject tumor in rechallenge experiment

As mice in complete remission from 4T1 and B16-F10 tumors remained tumor-free, we examined whether the systemic and local activation of T cells persisted and was sufficient to prevent a tumor from establishing in a tumor rechallenge.

**Fig. 7:**
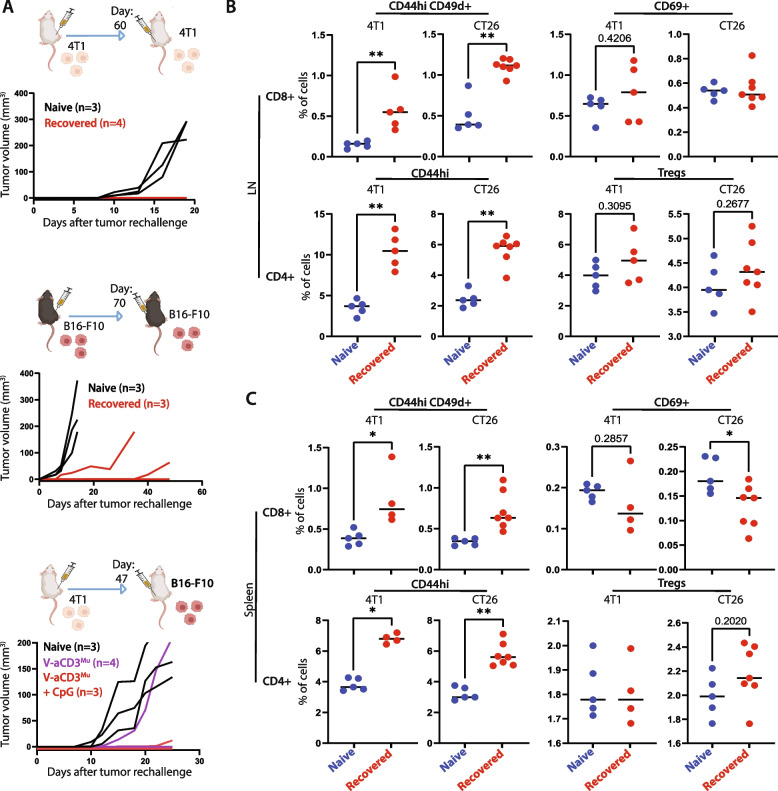
Recovered mice reject tumor in rechallenge experiment. **A** Left/middle: Tumor measurements of tumor-ablating BALB/c (white) and C57BL/6 (black) mice rechallenged with the same number of 4T1 or B16-F10 cancer cells, respectively, on either day 60 or 70 after the first tumor injection. Right: Tumor measurements of 4T1 tumor-ablating mice rechallenged with B16-F10 cancer cells on day 47 after the first tumor injection. Naïve mice were included in all rechallenges as controls for tumor take. **B **+** C** Flow cytometry on cell populations from spleens and LNs from either naïve mice (blue), or mice that had recovered from 4T1 or CT26 cancer (red). The percentage of cells represents the percentage of live cells within the lymphocyte gate. Differences between cell fractions in naive and survivor mice were evaluated using the Mann–Whitney test. *p < 0.05, **p < 0.01, ***p < 0.001, ****p < 0.0001

Mice recovered from 4T1 by V-aCD3^Mu^ combined with aCTLA-4 treatment were rechallenged in the opposite flank 60 days after the initial injection of cancer cells. None of the recovered mice developed tumors while all naïve mice challenged in parallel were susceptible (Fig. 7A). Mice that had successfully recovered from B16-F10 tumors after receiving V-aCD3^Mu^ and aCTLA-4 in combination were rechallenged on day 70. These mice were partially protected as one remained tumor-free and two out of three mice showed a delay in tumor growth.

To study whether this was tumor specific, 4T1 tumor-ablating BALB/c mice were rechallenged with B16-F10 tumors with a C57BL/6 background on day 49 after the initial tumor cell injection. The allogeneic cancer cells established tumors in the naïve control mice but were rejected in five out of seven mice recovered from 4T1 tumors after V-aCD3^Mu^ (three out of four mice) or V-aCD3^Mu^ with CpG treatment (two out of three mice). This suggests an immune response towards neoantigens shared between the two tumor types. We observed tumor cell-specific antibody induction (non-ofCS specific) after treatment but whether this aids in the cancer cell rejection remains to be elucidated (Fig. S7).

We next assessed the presence of activated and memory T cells in spleens and draining lymph nodes of recovered mice. Tissues were harvested 154 and 120 days after tumor cell injection of CT26 and 4T1, respectively, and naïve mice were included as negative controls. Compared to controls, the percentage of both T helper cell memory (CD4 + CD44^hi^) and antigen-exposed cytotoxic memory cells (CD8 + CD44^hi^CD49d +) were significantly higher in mice recovered from both tumor types in the LNs as well as in the spleens (Fig. 7B + C) [49]. Surprisingly, the percentage of activated/resident cytotoxic T cells was lower in spleens from recovered mice compared to naïve mice. The percentage of Tregs was comparable in mice treated for cancer and naïve controls. This points towards a T cell-driven neoantigen response.

In summary, treatment using V-aCD3^Mu^ in combination with an ICI provide long-lasting local and systemic T cell responses.

## Discussion

In this study, we demonstrate a strong anti-tumor effect of a bispecific molecule consisting of a binding domain from the malaria protein VAR2CSA recognizing a cancer-specific chondroitin sulfate and a scFv binding murine CD3 (V-aCD3^Mu^). V-aCD3^Mu^ both genetically fused and coupled using the SpyTag/SpyCatcher system effectively bound murine T cells and all tested cancer cells lines, similar to the binding previously described for rVAR2 [12]. V-aCD3^Mu^ mediated in vitro T cell-killing of cancer cells. In vivo, V-aCD3^Mu^ treatment prevented tumor development of 4T1 cancer cells and significantly delayed tumor growth in an established orthotopic pancreatic cancer model. V-aCD3^Mu^ combined with an ICI and/or CpG successfully eliminated established 4T1, B16-F10, and CT26 tumors, without increasing systemic cytokine levels further than ICIs alone. Notably, the kinetics were not identical between the models, which we speculate is attributed to differences in tumor growth, angiogenesis, tumor mutations, and breed of mice. The variable kinetics within each model could be due to a critical threshold of activated versus exhausted T-cells relative to tumor size needed to induce regression, but this needs to be further investigated. Recovered mice stayed tumor-free and were partially protected against tumor rechallenge more than 70 days after the first cancer cell injection.

Various glycosaminoglycans (hyaluronic acid, heparin) and proteoglycans (CD44, CSPG4, MUC-1) have been suggested as potential cancer targets due to their important roles in tumor pathogenesis and their upregulation in malignant tissues [14, 16, 18, 50, 51] but are also expressed in healthy tissues. This is also the case for chondroitin sulfate A in general. However, the distinct sulfation pattern on ofCS is exclusively expressed on the surface of a broad range of cancer cells and their surrounding extracellular matrix [12]. The ofCS-binding protein rVAR2 might therefore be the key to specifically target the changes in sulfation patterns within tumors.

T cell and NK cell-induced apoptosis of tumor cells has shown to be immunogenic and increase MHC-II presentation of tumor neoantigens [39]. We found that the therapeutic effect of V-aCD3^Mu^ and aCTLA-4 was accompanied by a systemic increase of both activated and memory T cells and an influx of memory T cells into the tumor, making the tumor immunologically “hot”. We also observed a pronounced increase in antigen-exposed memory T cells in the recovered mice more than 120 days after tumor inoculation. In addition, mice recovered from 4T1 tumors rejected rechallenge with B16-F10 tumors. This suggests that T cell activation towards tumor neoantigens, which can also be generated through post-translational modifications such as glycosylation, might be a driver of the observed immunogenicity and anti-tumor effects [52].

However, as tumor microenvironments are often immunosuppressive, tumor-specific T cells created after V-aCD3^Mu^ tumor cell killing might still be suppressed. We used CpG as proof-of-concept as it potently stimulates both innate and adaptive immunity. However, it has failed clinical trials due to lack of efficacy at safe dosage. Advanced delivery methods are likely needed for CpG to reach the clinic [53]. ICIs, on the other hand, have transformed the field of cancer immunotherapy by inhibiting immunosuppression, thereby providing new treatment options for cancer patients. But as immunologically “hot” tumors generally respond better to ICIs than immunologically “cold” tumors, a combination of a high mutational burden and a strong infiltration of T cells is required in the tumor for ICIs to exert their functions. New strategies, like combining ICI treatment with V-aCD3, can potentially help provide these optimal conditions to allow for therapeutic efficacy in many cancer patients currently lacking options [54–56].

Another challenge in prolonging the life of cancer patients is metastases, making a systemic anti-tumor response in the patients important for survival. Through the local administration of V-aCD3^Mu^, we observed abscopal anti-tumor effects of the combined V-aCD3^Mu^ and CpG treatment. This is in line with other studies also showing an abscopal effect after local administration [41, 57–60]. As V-aCD3 includes a recombinant malaria protein and no Fc-region it has a short half-life and is not ideal for intravenous administration. However, local administration might likely be advantageous compared to intravenous administration regarding efficacy due to epitope spreading, reduced side-effects, and hence higher tolerated dose. Although this excludes some patients where the tumor cannot be reached, others are included as side-effects are likely reduced [41].

Next steps for developing a clinical-grade compound for human testing will be to determine the efficacy and safety of the human-targeting V-aCD3 when combined with an ICI.

## Conclusion

In this study, we demonstrate how the bispecific construct V-aCD3^Mu^, which binds a unique glycosaminoglycan broadly expressed on cancer cells, works more potently in combination with different ICIs to make immunologically “cold” tumors “hot”. This results in complete elimination of established solid tumors. Together, this data highlights how combining ICIs with V-aCD3 might broaden the therapeutic potential of ICIs to include many patients currently without effective therapy.

## Supplementary Information

**Additional file 1: Sup. Fig. 1.** (A) ELISA showing binding of V-aCD3^Mu^ (Coupled)(Kd = 38.8, Bmax = 3.31), rVAR2 (Kd and Bmax not determined), and V-aCD3^Mu^ (Fused)(Kd = 14.2, Bmax = 3.36) to CSPG on a decorin backbone. Data is representative of a minimum of two separate experiments. (B) Solid 4T1 tumors 50-100 mm^3^ in size were treated with either PBS (*n*=5), V-aCD3^Mu^ (Coupled) + CpG (*n*=8), or V-aCD3^Mu^ (Fused) + CpG (*n*=8) on day 10, 12, 14, and 17 after tumor injection. Numbers in parentheses indicate the number of animals with complete tumor regression out of all mice in the group.** Sup. Fig. 2.** (A) Gating strategy on splenocytes and PBMCs in flow cytometry used to determine binding of rVAR2, aCD3^Mu^, V-aCD3^Mu^, aCD3^Hu^, and anti-V5 antibodies to T cells and non-T cell splenocytes/PBMCs. The gating is single cells lymphocytes live cells CD4+ and/or CD8+ cells as T cells and CD4-CD8- cells as non-T cells. The geometric MFI of the anti-penta-HIS antibodies conjugated to Alexa Flour 488 was then used to evaluate the binding of the HIS-tagged proteins. (B) Binding of aCD3^Mu^ (Kd = 4.96, Bmax = 1.05), rVAR2 (Kd = NR, Bmax = 0.46), and V-aCD3^Mu^ (Kd = 1.24, Bmax = 3.38) to murine recombinant CD3 in ELISA with aCD4^Mu^ as a negative control (left). Means and standard deviations are shown. Right pane shows CSA inhibition of binding at 120 nM (right). Each dot represents one data point. **Sup. Fig. 3.** Cytokines measured from 4T1 and splenocyte co-culture supernatants using ELISA. Mouse splenocytes were incubated with 4T1 cancer cells together with 200 nM of the indicated protein. **Sup. Fig. 4.** (A) Survival curves for mice with indicated tumors treated as described in Fig. 4. The cut-off for all Kaplan-Meier plots is a tumor volume of $\ge$ 400 mm^3^. Mice were censored if they had to be excluded from the study prematurely due to reasons other than tumor size. Log-rank test was used for statistical analysis. *p < 0.05. (B) Bioluminescence in vivo imaging of C57BL/6 mice following orthotopic implantation of 5x10^4^ Luciferase^+^ primary pancreatic cancer cells (CHX2000) derived from KPC mice (LSL-Kras^G12D/+^; p53^f/f^; Pdx1-Cre).** Sup. Fig. 5. **(A-C) Survival curves for mice treated as described in Fig. 5. The cut-off for all Kaplan-Meier plots is a tumor volume of $\ge$ 400 mm^3^. Mice were censored if they had to be excluded from the study prematurely due to reasons other than tumor size. Log-rank test was used for statistical analysis. *p < 0.05, **p < 0.01, ***p < 0.001, ****p < 0.0001.** Sup. Fig. 6**. (A) Treatment schedule until day 14 when spleens and tumors were harvested for flow cytometry and the subsequent gating strategy on splenocytes to evaluate different cell types in C-D. (B) Percentage of live cells relative to the PBS group in the spleen. Both CD8+ and CD4+ T cells that are CD69+, CD44hi, CD8+CD25+, or Tregs are shown. (C) UMAPs of splenocytes from all four treatment groups with clustering performed in ClusterExplorer. Cell types in clusters are explained below. Statistics were performed using one-way ANOVA with Dunnett’s post hoc test for comparison of all treatment groups to the PBS group. *P* values are indicated if significant or important for reading the figure. (D) Correlations between the tumor size and %CD8+CD69+ (*p*=0.67) and %CD4+CD69+ (*p*=0.14) of all live single cells in the tumor evaluated by simple linear regression.** Sup. Fig. 7.** Binding of mouse antibodies to 4T1 cells and B16-F10 cells in flow cytometry. Serum from C57BL/6 mice treated as described in materials and methods was diluted as illustrated on the figure and incubated with 200.000 4T1 or B16-F10 cells. Soluble CSA was added if indicated for 1 hour before detection with an anti-mouse IgG antibody conjugated to FITC.

## Data Availability

The datasets used and/or analyzed during the current study are available from the corresponding author on reasonable request.

## Abbreviations

V-aCD3^Hu^: Human T cell-engager targeting CD3 and oncofetal chondroitin sulfate
V-aCD3^Mu^: Recombinant VAR2CSA linked to a single chain-variable fragment targeting murine anti-CD3
TAAs: Tumor-associated antigens
T-bsAbs: T cell-engaging bispecific antibodies
rVAR2: Recombinant VAR2CSA
PBMCs: Peripheral blood mononuclear cells
ofCS: Oncofetal chondroitin sulfate
aCD3^Mu^: Anti-murine CD3 single-chain variable fragment
CpG: CpG-oligodeoxynucleotides
ICI: Immune checkpoint inhibitor
SQ: Subcutaneously
CSA: Chondroitin sulfate A
aPD-1: Anti-PD-1
aCTLA-4: Anti-CTLA-4
CSPG: Chondroitin sulfate proteoglycan
CS: Chondroitin sulfate
LNs: Lymph nodes
E:T: Effector-target ratio
FBS: Fetal bovine serum
