# Supplementary material for: Bispecific T cell-engager targeting oncofetal chondroitin sulfate induces complete tumor regression and protective immune memory in mice
Source: J Exp Clin Cancer Res. 2023 Apr 28;42:106. doi: 10.1186/s13046-023-02655-8 (PMC10142489; doi:10.1186/s13046-023-02655-8)
Supplement: Supplementary file 1 — Additional file 1: Sup. Fig. 1. (A) ELISA showing binding of V-aCD3Mu (Coupled)(Kd = 38.8, Bmax = 3.31), rVAR2 (Kd and Bmax not determined), and V-aCD3Mu (Fused)(Kd = 14.2, Bmax = 3.36) to CSPG on a decorin backbone. Data is representative of a minimum of two separate experiments. (B) Solid 4T1 tumors 50-100 mm3 in size were treated with either PBS (n=5), V-aCD3Mu (Coupled) + CpG (n=8), or V-aCD3Mu (Fused) + CpG (n=8) on day 10, 12, 14, and 17 after tumor injection. Numbers in parentheses indicate the number of animals with complete tumor regression out of all mice in the group. Sup. Fig. 2. (A) Gating strategy on splenocytes and PBMCs in flow cytometry used to determine binding of rVAR2, aCD3Mu, V-aCD3Mu, aCD3Hu, and anti-V5 antibodies to T cells and non-T cell splenocytes/PBMCs. The gating is single cells lymphocytes live cells CD4+ and/or CD8+ cells as T cells and CD4-CD8- cells as non-T cells. The geometric MFI of the anti-penta-HIS antibodies conjugated to Alexa Flour 488 was then used to evaluate the binding of the HIS-tagged proteins. (B) Binding of aCD3Mu (Kd = 4.96, Bmax = 1.05), rVAR2 (Kd = NR, Bmax = 0.46), and V-aCD3Mu (Kd = 1.24, Bmax = 3.38) to murine recombinant CD3 in ELISA with aCD4Mu as a negative control (left). Means and standard deviations are shown. Right pane shows CSA inhibition of binding at 120 nM (right). Each dot represents one data point. Sup. Fig. 3. Cytokines measured from 4T1 and splenocyte co-culture supernatants using ELISA. Mouse splenocytes were incubated with 4T1 cancer cells together with 200 nM of the indicated protein. Sup. Fig. 4. (A) Survival curves for mice with indicated tumors treated as described in Fig. 4. The cut-off for all Kaplan-Meier plots is a tumor volume of $\ge$≥ 400 mm3. [file 13046_2023_2655_MOESM1_ESM.zip › 13046_2023_2655_MOESM1_ESM.docx]

**SUPPLEMENTARY METHODS**

**ELISA assays for binding to CSPG on a decorin backbone**

Falcon 96 well plates (BD Biosciences) were coated with either 150 ng human decorin per well (Proteogenix), HSPG (Sigma-Aldrich), or TSM binding/blocking buffer (TSM buffer + 0.05% Tween20 + 1% BSA) overnight at 4ᵒC. The wells were then blocked for 1-2 hours with TSM blocking buffer. Samples were then added to the wells in TSM binding buffer and incubated for one hour at 37ᵒC. After washing in TSM buffer + 0,05% tween three times, the plates were incubated one hour at 37ᵒC with either anti-V5-HRP antibody (Abcam, 1:9000) or anti-penta-HIS-HRP (Qiagen, 1:3000) in TSM binding buffer. When using the anti-penta-HIS antibody, an additional incubation with anti-mouse-HRP (Dako, 1:3000) was performed. The plates were developed using TMB-Plus (Kementec) and the absorbance at 450 nm was measured using a HiPo MPP-96 microplate photometer.

**ELISA assays for binding to murine CD3**

MaxiSorp plates (ThermoFisher) were coated with recombinant murine CD3 epsilon (0.1 mg/mL, SinoBiological) overnight at 4ᵒC. PBS + 0.05% Tween20 (PBS-T) was used for washing before 1-2 hours blocking using 5% skimmed milk in PBS-T. Protein was added either alone or together with CSA and incubated for 1 hour at RT and subsequently detected using anti-penta-HIS-HRP or goat anti-rat-HRP (Abcam, 1:3000). The plates were developed as described above.

**Detection of antibodies bound to cancer cells**

Antibody binding to cancer cells from mouse serum was evaluated in flow cytometry. Mouse serum was frozen down, on a later day thawed, and incubated for 1 hour with either 4T1 or B16-F10 cells. Subsequently, the bound antibodies were detected with anti-mouse IgG (H+L, Fluorescein) from Vector Laboratories.

**Cytokine measurements from supernatants**

Cytokine levels in the supernatants were measured by ELISA using MaxiSorp plates (ThermoFisher) and ELISA MAX™ Deluxe Set Mouse for detection of IL-2, IFN-γ, and TNF-α (Biolegend), following the manufacturer’s instructions. Absorbance was measured using the HiPo MPP-96 microplate photometer. The absorbance at 570 nm was subtracted the absorbance at 450 nm.

**Kaplan-Meier curves**

Kaplan-Meier curves were made with a cut-off of $\geq400$ mm3 and Log-rank test was used for statistical analysis.

**UMAPs**

Plugins for FlowJo were used for generating UMAPs (v3.1) and clustering (ClusterExplorer, v1.6.5).

**SUPPLEMENTARY FIGURES**

**Sup. Fig. 1.** (**A**) ELISA showing binding of V-aCD3^Mu^ (Coupled)(Kd = 38.8, Bmax = 3.31), rVAR2 (Kd and Bmax not determined), and V-aCD3^Mu^ (Fused)(Kd = 14.2, Bmax = 3.36) to CSPG on a decorin backbone. Data is representative of a minimum of two separate experiments. (**B**) Solid 4T1 tumors 50-100 mm^3^ in size were treated with either PBS (n=5), V-aCD3^Mu^ (Coupled) + CpG (n=8), or V-aCD3^Mu^ (Fused) + CpG (n=8) on day 10, 12, 14, and 17 after tumor injection. Numbers in parentheses indicate the number of animals with complete tumor regression out of all mice in the group.

**Sup. Fig. 2.** (**A**) Gating strategy on splenocytes and PBMCs in flow cytometry used to determine binding of rVAR2, aCD3^Mu^, V-aCD3^Mu^, aCD3^Hu^, and anti-V5 antibodies to T cells and non-T cell splenocytes/PBMCs. The gating is single cells 🡪 lymphocytes 🡪 live cells 🡪 CD4+ and/or CD8+ cells as T cells and CD4-CD8- cells as non-T cells. The geometric MFI of the anti-penta-HIS antibodies conjugated to Alexa Flour 488 was then used to evaluate the binding of the HIS-tagged proteins. (**B**) Binding of aCD3^Mu^ (Kd = 4.96, Bmax = 1.05), rVAR2 (Kd = NR, Bmax = 0.46), and V-aCD3^Mu^ (Kd = 1.24, Bmax = 3.38) to murine recombinant CD3 in ELISA with aCD4^Mu^ as a negative control (left). Means and standard deviations are shown. Right pane shows CSA inhibition of binding at 120 nM (right). Each dot represents one data point.

**Sup. Fig. 3.** Cytokines measured from 4T1 and splenocyte co-culture supernatants using ELISA. Mouse splenocytes were incubated with 4T1 cancer cells together with 200 nM of the indicated protein.

**Sup. Fig. 4.** (**A**) Survival curves for mice with indicated tumors treated as described in Fig. 4. The cut-off for all Kaplan-Meier plots is a tumor volume of $\geq$ 400 mm^3^. Mice were censored if they had to be excluded from the study prematurely due to reasons other than tumor size. Log-rank test was used for statistical analysis. **p* < 0.05. (**B**) Bioluminescence in vivo imaging of C57BL/6 mice following orthotopic implantation of 5x10^4^ Luciferase^+^ primary pancreatic cancer cells (CHX2000) derived from KPC mice (LSL-Kras^G12D/+^; p53^f/f^; Pdx1-Cre).

**Sup. Fig. 5.** (**A-C**) Survival curves for mice treated as described in Fig. 5. The cut-off for all Kaplan-Meier plots is a tumor volume of $\geq$ 400 mm^3^. Mice were censored if they had to be excluded from the study prematurely due to reasons other than tumor size. Log-rank test was used for statistical analysis. **p* < 0.05, ***p* < 0.01, ****p* < 0.001, *****p* < 0.0001.

**Sup. Fig. 6**. (**A**) Treatment schedule until day 14 when spleens and tumors were harvested for flow cytometry and the subsequent gating strategy on splenocytes to evaluate different cell types in C-D. (**B**) Percentage of live cells relative to the PBS group in the spleen. Both CD8+ and CD4+ T cells that are CD69+, CD44hi, CD8+CD25+, or Tregs are shown. (**C**) UMAPs of splenocytes from all four treatment groups with clustering performed in ClusterExplorer. Cell types in clusters are explained below. Statistics were performed using one-way ANOVA with Dunnett’s post hoc test for comparison of all treatment groups to the PBS group. *P* values are indicated if significant or important for reading the figure.

(**D**) Correlations between the tumor size and %CD8+CD69+ (*p*=0.67) and %CD4+CD69+ (*p*=0.14) of all live single cells in the tumor evaluated by simple linear regression.

**Sup. Fig. 7.** Binding of mouse antibodies to 4T1 cells and B16-F10 cells in flow cytometry. Serum from C57BL/6 mice treated as described in materials and methods was diluted as illustrated on the figure and incubated with 200.000 4T1 or B16-F10 cells. Soluble CSA was added if indicated for 1 hour before detection with an anti-mouse IgG antibody conjugated to FITC.
