# Supplementary figures and images for: Bispecific T cell-engager targeting oncofetal chondroitin sulfate induces complete tumor regression and protective immune memory in mice
Source: J Exp Clin Cancer Res. 2023 Apr 28;42:106. doi: 10.1186/s13046-023-02655-8 (PMC10142489; doi:10.1186/s13046-023-02655-8)

A

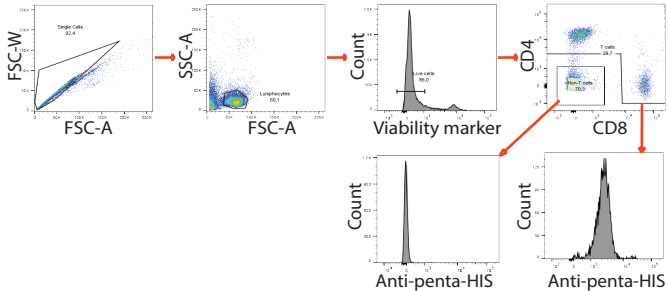

B

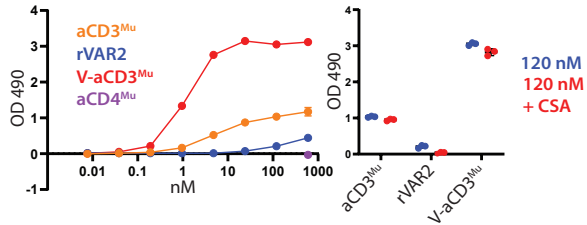

Supplement: Supplementary file 1 — Additional file 1: Sup. Fig. 1. (A) ELISA showing binding of V-aCD3Mu (Coupled)(Kd = 38.8, Bmax = 3.31), rVAR2 (Kd and Bmax not determined), and V-aCD3Mu (Fused)(Kd = 14.2, Bmax = 3.36) to CSPG on a decorin backbone. Data is representative of a minimum of two separate experiments. (B) Solid 4T1 tumors 50-100 mm3 in size were treated with either PBS (n=5), V-aCD3Mu (Coupled) + CpG (n=8), or V-aCD3Mu (Fused) + CpG (n=8) on day 10, 12, 14, and 17 after tumor injection. Numbers in parentheses indicate the number of animals with complete tumor regression out of all mice in the group. Sup. Fig. 2. (A) Gating strategy on splenocytes and PBMCs in flow cytometry used to determine binding of rVAR2, aCD3Mu, V-aCD3Mu, aCD3Hu, and anti-V5 antibodies to T cells and non-T cell splenocytes/PBMCs. The gating is single cells lymphocytes live cells CD4+ and/or CD8+ cells as T cells and CD4-CD8- cells as non-T cells. The geometric MFI of the anti-penta-HIS antibodies conjugated to Alexa Flour 488 was then used to evaluate the binding of the HIS-tagged proteins. (B) Binding of aCD3Mu (Kd = 4.96, Bmax = 1.05), rVAR2 (Kd = NR, Bmax = 0.46), and V-aCD3Mu (Kd = 1.24, Bmax = 3.38) to murine recombinant CD3 in ELISA with aCD4Mu as a negative control (left). Means and standard deviations are shown. Right pane shows CSA inhibition of binding at 120 nM (right). Each dot represents one data point. Sup. Fig. 3. Cytokines measured from 4T1 and splenocyte co-culture supernatants using ELISA. Mouse splenocytes were incubated with 4T1 cancer cells together with 200 nM of the indicated protein. Sup. Fig. 4. (A) Survival curves for mice with indicated tumors treated as described in Fig. 4. The cut-off for all Kaplan-Meier plots is a tumor volume of $\ge$≥ 400 mm3. [file 13046_2023_2655_MOESM1_ESM.zip › Fig S2.pdf]

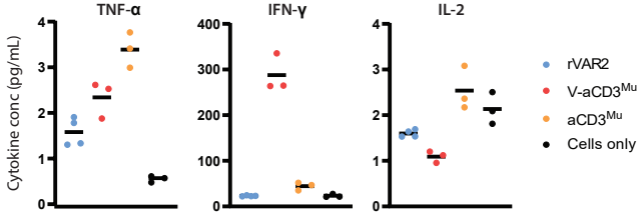

Supplement: Supplementary file 1 — Additional file 1: Sup. Fig. 1. (A) ELISA showing binding of V-aCD3Mu (Coupled)(Kd = 38.8, Bmax = 3.31), rVAR2 (Kd and Bmax not determined), and V-aCD3Mu (Fused)(Kd = 14.2, Bmax = 3.36) to CSPG on a decorin backbone. Data is representative of a minimum of two separate experiments. (B) Solid 4T1 tumors 50-100 mm3 in size were treated with either PBS (n=5), V-aCD3Mu (Coupled) + CpG (n=8), or V-aCD3Mu (Fused) + CpG (n=8) on day 10, 12, 14, and 17 after tumor injection. Numbers in parentheses indicate the number of animals with complete tumor regression out of all mice in the group. Sup. Fig. 2. (A) Gating strategy on splenocytes and PBMCs in flow cytometry used to determine binding of rVAR2, aCD3Mu, V-aCD3Mu, aCD3Hu, and anti-V5 antibodies to T cells and non-T cell splenocytes/PBMCs. The gating is single cells lymphocytes live cells CD4+ and/or CD8+ cells as T cells and CD4-CD8- cells as non-T cells. The geometric MFI of the anti-penta-HIS antibodies conjugated to Alexa Flour 488 was then used to evaluate the binding of the HIS-tagged proteins. (B) Binding of aCD3Mu (Kd = 4.96, Bmax = 1.05), rVAR2 (Kd = NR, Bmax = 0.46), and V-aCD3Mu (Kd = 1.24, Bmax = 3.38) to murine recombinant CD3 in ELISA with aCD4Mu as a negative control (left). Means and standard deviations are shown. Right pane shows CSA inhibition of binding at 120 nM (right). Each dot represents one data point. Sup. Fig. 3. Cytokines measured from 4T1 and splenocyte co-culture supernatants using ELISA. Mouse splenocytes were incubated with 4T1 cancer cells together with 200 nM of the indicated protein. Sup. Fig. 4. (A) Survival curves for mice with indicated tumors treated as described in Fig. 4. The cut-off for all Kaplan-Meier plots is a tumor volume of $\ge$≥ 400 mm3. [file 13046_2023_2655_MOESM1_ESM.zip › Fig S3.pdf]

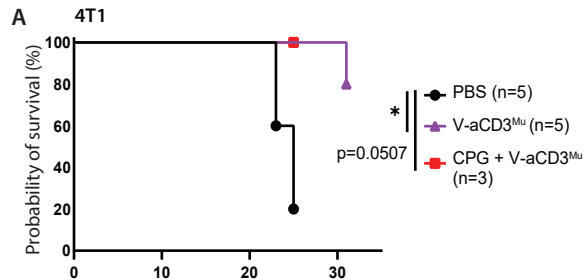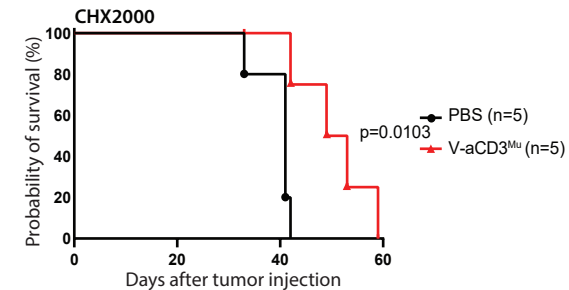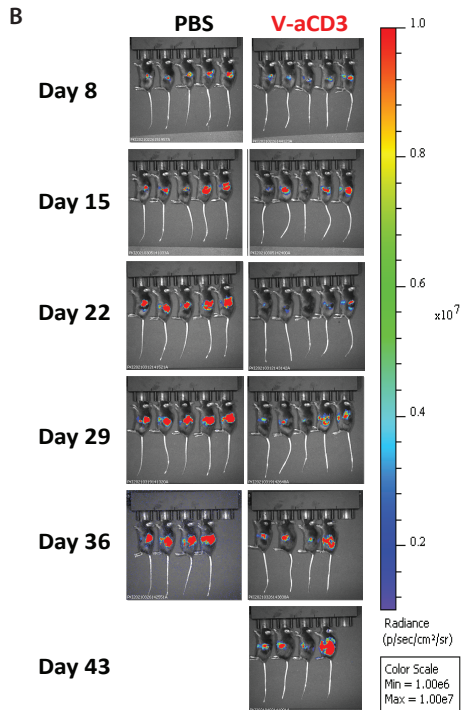

Supplement: Supplementary file 1 — Additional file 1: Sup. Fig. 1. (A) ELISA showing binding of V-aCD3Mu (Coupled)(Kd = 38.8, Bmax = 3.31), rVAR2 (Kd and Bmax not determined), and V-aCD3Mu (Fused)(Kd = 14.2, Bmax = 3.36) to CSPG on a decorin backbone. Data is representative of a minimum of two separate experiments. (B) Solid 4T1 tumors 50-100 mm3 in size were treated with either PBS (n=5), V-aCD3Mu (Coupled) + CpG (n=8), or V-aCD3Mu (Fused) + CpG (n=8) on day 10, 12, 14, and 17 after tumor injection. Numbers in parentheses indicate the number of animals with complete tumor regression out of all mice in the group. Sup. Fig. 2. (A) Gating strategy on splenocytes and PBMCs in flow cytometry used to determine binding of rVAR2, aCD3Mu, V-aCD3Mu, aCD3Hu, and anti-V5 antibodies to T cells and non-T cell splenocytes/PBMCs. The gating is single cells lymphocytes live cells CD4+ and/or CD8+ cells as T cells and CD4-CD8- cells as non-T cells. The geometric MFI of the anti-penta-HIS antibodies conjugated to Alexa Flour 488 was then used to evaluate the binding of the HIS-tagged proteins. (B) Binding of aCD3Mu (Kd = 4.96, Bmax = 1.05), rVAR2 (Kd = NR, Bmax = 0.46), and V-aCD3Mu (Kd = 1.24, Bmax = 3.38) to murine recombinant CD3 in ELISA with aCD4Mu as a negative control (left). Means and standard deviations are shown. Right pane shows CSA inhibition of binding at 120 nM (right). Each dot represents one data point. Sup. Fig. 3. Cytokines measured from 4T1 and splenocyte co-culture supernatants using ELISA. Mouse splenocytes were incubated with 4T1 cancer cells together with 200 nM of the indicated protein. Sup. Fig. 4. (A) Survival curves for mice with indicated tumors treated as described in Fig. 4. The cut-off for all Kaplan-Meier plots is a tumor volume of $\ge$≥ 400 mm3. [file 13046_2023_2655_MOESM1_ESM.zip › Fig S4.pdf]

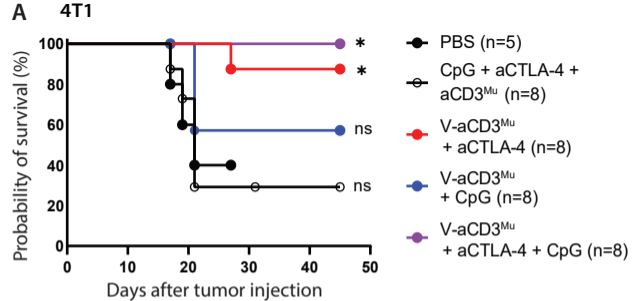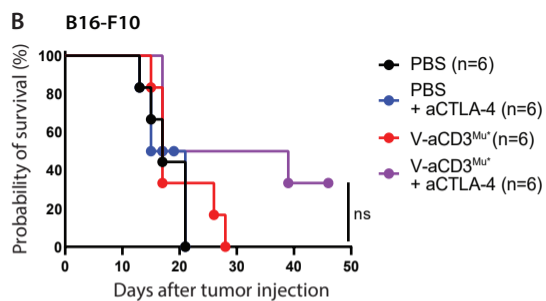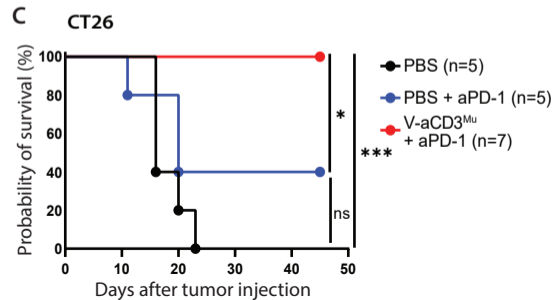

Supplement: Supplementary file 1 — Additional file 1: Sup. Fig. 1. (A) ELISA showing binding of V-aCD3Mu (Coupled)(Kd = 38.8, Bmax = 3.31), rVAR2 (Kd and Bmax not determined), and V-aCD3Mu (Fused)(Kd = 14.2, Bmax = 3.36) to CSPG on a decorin backbone. Data is representative of a minimum of two separate experiments. (B) Solid 4T1 tumors 50-100 mm3 in size were treated with either PBS (n=5), V-aCD3Mu (Coupled) + CpG (n=8), or V-aCD3Mu (Fused) + CpG (n=8) on day 10, 12, 14, and 17 after tumor injection. Numbers in parentheses indicate the number of animals with complete tumor regression out of all mice in the group. Sup. Fig. 2. (A) Gating strategy on splenocytes and PBMCs in flow cytometry used to determine binding of rVAR2, aCD3Mu, V-aCD3Mu, aCD3Hu, and anti-V5 antibodies to T cells and non-T cell splenocytes/PBMCs. The gating is single cells lymphocytes live cells CD4+ and/or CD8+ cells as T cells and CD4-CD8- cells as non-T cells. The geometric MFI of the anti-penta-HIS antibodies conjugated to Alexa Flour 488 was then used to evaluate the binding of the HIS-tagged proteins. (B) Binding of aCD3Mu (Kd = 4.96, Bmax = 1.05), rVAR2 (Kd = NR, Bmax = 0.46), and V-aCD3Mu (Kd = 1.24, Bmax = 3.38) to murine recombinant CD3 in ELISA with aCD4Mu as a negative control (left). Means and standard deviations are shown. Right pane shows CSA inhibition of binding at 120 nM (right). Each dot represents one data point. Sup. Fig. 3. Cytokines measured from 4T1 and splenocyte co-culture supernatants using ELISA. Mouse splenocytes were incubated with 4T1 cancer cells together with 200 nM of the indicated protein. Sup. Fig. 4. (A) Survival curves for mice with indicated tumors treated as described in Fig. 4. The cut-off for all Kaplan-Meier plots is a tumor volume of $\ge$≥ 400 mm3. [file 13046_2023_2655_MOESM1_ESM.zip › Fig S5.pdf]

Serum anti-4T1 antibodies

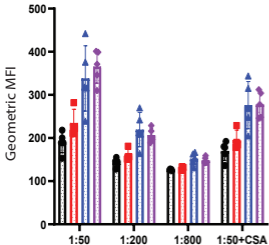

Serum anti-B16-F10 antibodies

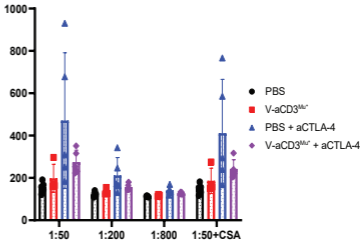

Supplement: Supplementary file 1 — Additional file 1: Sup. Fig. 1. (A) ELISA showing binding of V-aCD3Mu (Coupled)(Kd = 38.8, Bmax = 3.31), rVAR2 (Kd and Bmax not determined), and V-aCD3Mu (Fused)(Kd = 14.2, Bmax = 3.36) to CSPG on a decorin backbone. Data is representative of a minimum of two separate experiments. (B) Solid 4T1 tumors 50-100 mm3 in size were treated with either PBS (n=5), V-aCD3Mu (Coupled) + CpG (n=8), or V-aCD3Mu (Fused) + CpG (n=8) on day 10, 12, 14, and 17 after tumor injection. Numbers in parentheses indicate the number of animals with complete tumor regression out of all mice in the group. Sup. Fig. 2. (A) Gating strategy on splenocytes and PBMCs in flow cytometry used to determine binding of rVAR2, aCD3Mu, V-aCD3Mu, aCD3Hu, and anti-V5 antibodies to T cells and non-T cell splenocytes/PBMCs. The gating is single cells lymphocytes live cells CD4+ and/or CD8+ cells as T cells and CD4-CD8- cells as non-T cells. The geometric MFI of the anti-penta-HIS antibodies conjugated to Alexa Flour 488 was then used to evaluate the binding of the HIS-tagged proteins. (B) Binding of aCD3Mu (Kd = 4.96, Bmax = 1.05), rVAR2 (Kd = NR, Bmax = 0.46), and V-aCD3Mu (Kd = 1.24, Bmax = 3.38) to murine recombinant CD3 in ELISA with aCD4Mu as a negative control (left). Means and standard deviations are shown. Right pane shows CSA inhibition of binding at 120 nM (right). Each dot represents one data point. Sup. Fig. 3. Cytokines measured from 4T1 and splenocyte co-culture supernatants using ELISA. Mouse splenocytes were incubated with 4T1 cancer cells together with 200 nM of the indicated protein. Sup. Fig. 4. (A) Survival curves for mice with indicated tumors treated as described in Fig. 4. The cut-off for all Kaplan-Meier plots is a tumor volume of $\ge$≥ 400 mm3. [file 13046_2023_2655_MOESM1_ESM.zip › Fig S7.pdf]
